# The clearance effect of bovine anti-*Helicobacter pylori* antibody-containing milk in O blood group *Helicobacter pylori*-infected patients: a randomized double-blind clinical trial

**DOI:** 10.1186/s12967-015-0558-1

**Published:** 2015-06-30

**Authors:** Dailun Hu, Feng Zhang, Jikun Zhou, Baohong Xu, Hongying Zhang, Huiqin Qiang, Shuguang Ren, Baoen Shan, Changfu Yin, Zhitao Zhang, Xian Wang, Chuan Zhao, Zhongli Shi

**Affiliations:** Clinical Department, The Research Section of Experimentation Teaching Center, Hebei Medical University, Shijiazhuang, People’s Republic of China; Shijiazhuang Center for Disease Control and Prevention, Shijiazhuang, People’s Republic of China; The Institute of Cereal and Oil Crop, Hebei Academy of Agriculture and Forestry Sciences, Shijiazhuang, People’s Republic of China; The Fourth Hospital of Hebei Medical University, Shijiazhuang, People’s Republic of China; Shijiazhuang Center for Prevention and Control of Animal Diseases, Shijiazhuang, People’s Republic of China

**Keywords:** *H. pylori*, Clearance rate, Cow milk, Antibodies, O blood group, Lewis blood group

## Abstract

**Background:**

The failure in standard triple therapy has recently increased to high levels in China, primarily because of insufficient patient compliance, antimicrobial resistance, and high costs. Effective prevention and eradication of *Helicobacter pylori* (*H. pylori*) by artificial passive immunization with orally administered bovine antibodies in the milk has been demonstrated in many animal studies, but the clinical studies that are available have shown no *H. pylori* eradication. This study was to evaluate the efficacy and safety of orally administered bovine anti-*H. pylori* antibodies for the clearance of *H. pylori* infecting O blood group subpopulations.

**Methods:**

Two local epidemic *H. pylori* strains that were prevalent locally were screened and then used to immunize dairy cows. After confirmation of the presence of anti-*H. pylori* polyclonal antibodies in the milk by enzyme-linked immunosorbent assay, the milk was subsequently defatted and processed into sterile milk by pasteurization. This study was designed as a double-blind placebo-controlled randomized clinical trial. Our 61 *H. pylori*-infected O blood group subjects were assigned to two groups; 31 subjects were treated with bovine milk containing antibodies and 30 subjects with the placebo. The medication-based study was continued for 28 days. Subjects were followed up for 56 days. The effect was assessed by the C-14 urea breath test (UBT). SPSS 17.0 software for Windows was used to analyze the data.

**Results:**

Of the 61 subjects enrolled, 58 completed the protocol. One volunteer in the antibodies group and two volunteers in the control group dropped out. Of the 30 antibody-treated subjects, 13 became UBT negative, whereas none of the 30 of the placebo-treated subjects became UBT negative after the medication. Of 13 UBT negative patients, 3 became positive again at the end of the follow-up. Both intention to treat and per-protocol analysis indicated a significant difference in the clearance rate of infected patients between the groups treated with bovine antibody-containing milk and the placebo (P = 0.001, P < 0.05) and no significant difference in adverse effects (P > 0.05 all).

**Conclusions:**

Bovine antibody-based oral immunotherapy appears to be safe and has a significant clearance effect on intragastric *H. pylori* that infects O blood group adults.

Trial registration: ChiCTR-TRC-14005212.

## Background

*Helicobacter pylori* infection is the main cause of gastritis, gastroduodenal ulcer, gastric adenocarcinoma, and mucosa-associated tissue lymphoma [1]. Indeed, *H. pylori* was recognized as a category I human carcinogen by the WHO/International Agency for Research on Cancer (WHO/IARC) in 1994 [2]. The eradication of *H. pylori* is now clearly central to the management of the illnesses described above.

The genotypes of *H. pylori* are based on two virulent genes: *cag*A and *vac*A. Different geographic locations have different *H. pylori* genotypes [3]. *Cag*A-positive *H. pylori* is reported to be 60–70% of *H. pylori* strains isolated from Europe, whereas more than 90% of *H. pylori* strains are *cag*A positive in eastern Asian countries [4, 5]. The *vac*A gene has *vac*A signal (s) and middle (m) regions, which are divided into s1 or s2 and m1 or m2, respectively. The *vac*A s1 region is further divided into s1a, s1b, and s1c. The m1 region is further classified into m1a, m1b, and m1c, and the m2 region into m2a and m2b [6, 7]. Different geographic regions have different combination of *vacA* allele and *cagA*. These variations in the global distribution of the *cag*A and *vac*A genotypes might account for the diversity of reports associating the *cag*A and *vac*A genotypes with the clinical outcome from these different regions. The serotypes of *H. pylori* are based on the different repeating oligosaccharides units of lipopolysacchride (LPS) molecules, whereby six distinct *H. pylori* serotypes (O1–O6) have been defined [8]. Subsequently, detailed structural studies have shown that the LPSs of *H. pylori* are unique in that their O-chain region is homologous to mammalian histo-blood group antigens. According to Lewis epitopes present in O-glycans and the terminal ABH structures, O-chain components of *H. pylori* are divided into type 1 Le^a^, Le^b^, Le^d^, Linear B and A determinants; type 2 *N*-acetyl-lactosamine (LacNAc), Lewis^x^ (Le^x^), Le^y^, Sialyl Le^x^, in monomeric and polymeric forms [9]. Like the genotype, the Lewis epitope of *H. pylori* has geographic variations. *H. pylori* strains from Asian population seem to have a higher tendency to produce the type 1 antigens, Le^a^ and Le^b^, whereas those isolated from North American and European hosts express mainly the type 2 antigens, Le^x^ and Le^y^. Some of *H. pylori* attach to the epithelium via BabA, SabA, and probably other adhesins. Epidemiologically, individuals of the O blood group are particularly prone to *H. pylori* infection because their gastric epithelium highly expresses the Le^b^ antigen, which is the BabA of *H. pylori* binding sites [10, 11].

The eradication rate after standard triple therapy has declined below 80% in most areas and has many adverse effects [12–14]. With the widespread use of antibiotics, the increased resistance to antibiotics and their expensive costs make eradication difficult in China [15]. New therapeutic strategies are needed to solve the problems mentioned above. The approach of passive immunization with orally administered antibodies against *H. pylori* is likely to constitute one of the new therapeutic strategies [16]. This approach mimics naïve protection in humans and has been shown to be effective in the prevention and treatment of a variety of pathogens, such as Rotavirus, *Clostridium difficile*, and *Campylobacter jejuni* [17–21]. Many animal studies have shown that bovine antibody-containing milk against *H. pylori* reduces bacterial load, thus preventing and even eradicating *H. pylori* infection [22–25]. However, the available clinical studies of bovine antibodies in milk have not shown *H. pylori* eradication [26–29]. Few clinical studies have been reported based on screening the local epidemic *H. pylori* strains according to Lewis blood group structure and the *vac*A allele to immunize diary cows. This clinical study was therefore performed to evaluate the efficacy and safety of specific anti-*H. pylori* polyclonal bovine antibodies in milk for the clearance of intragastric *Helicobacter* infection in O blood group subpopulations.

## Methods

### Bacterial strains and growth

Two local epidemic strains in Shijiazhuang city were screened by sequencing the *vac*A allele and *Cag*A gene (Table 1) of a total of 276 *H. pylori* strains that were isolated and collected between March 2008 and August 2011 from the gastric antrum or corpus of patients who had undergone endoscopy at the Fourth Hospital of Hebei Medical University [30, 31]. The two *H. pylori* strains were named Hp5162 and Hp4236, and the *vac*A genotype was respectively s1a/m2 (127/276) and s1a/m1 (87/276), both of which were *Cag*A-positive and isolated from O blood group patients. The phenotype of the Lewis blood-group antigens in the LPS of two *H. pylori* strains was analyzed by serological studies, by using Lewis blood-group-specific Le^a^, Le^b^, Le^x^, Le^y^ monoclonal antibodies (Santa Cruz Biotechnology Inc, Dallas, TX, USA) on whole cells by enzyme-linked immunosorbent assay (ELISA), respectively, with both indicating the presence of the type-1 Le^b^ epitope on the cell surface [32, 33]. Two *H. pylori* strains, which were preserved as frozen stocks at −80°C in brain heart infusion media supplemented with 20% glycerol and 10% fetal bovine serum (FBS), were revived and cultured on Columbia Agar medium plates (Oxoid Ltd., Basingstoke, Hampshire, England) supplemented with 10% defibrinated sheep blood, 5 mg/L trimethoprim, 5 mg/L polymixin-B, 5 mg/L amphoteracin-B, and 10 mg/L vancomucin (all antibiotics were purchased from Solarbio, Beijing, China), under micro-aerobic conditions (10% CO_2_, 85% N_2_, 5% O_2_) at 37°C for 3–10 days with 95% humidity. *H. pylori* bacterial cells were harvested from 9-cm Agar plates with a sterile loop. The *H. pylori* bacterial cells were washed with sterile 0.9% normal saline three times, suspended in 0.1 mol/L phosphate-buffered saline (PBS) for use as vaccines, and kept at −80°C until required.

**Table 1:** Oligonucleotide primers used for polymerase chain reaction (PCR), PCR conditions, and product size of *vac*A alleles and *cag*A

Genes	Primers	Product size (bp)	Initial denaturation temperature °C (min)	Denaturation temperature °C (min)	Annealing temperature °C (min)	Extension temperature °C (min)	Cycles	Final extension temperature °C (min)
s1a	F:5′-GTCAGCATCACACCGCAAC-3′	190		94 (1)	52 (1)	72 (1)	35	72 (5)
	R:5′-CTGCTTGAATGCGCCAAAC-3′							
s1b	F:5′-AGCGCCATACCGCAAGAG-3′	187		94 (1)	52 (1)	72 (1)	35	72 (5)
	R:5′-CTGCTTGAATGCGCCAAAC-3′							
s2	F: 5′-GCTAACACGCCAAATGATCC-3′	199		94 (1)	52 (1)	72 (1)	35	72 (5)
	R: 5′-CTGCTTGAATGCGCCAAAC-3′							
m1a	F: 5′-GGTCAAAATGCGGTCATGG-3′	290	95 (2)	94 (0.5)	52 (0.5)	72 (0.5)	40	72 (5)
	R: 5′-CCATTGGTACCTGTAGAAAC-3′							
m1b	F: 5′-GGCCCCAATGCAGTCATGGAT-3′	240–270	95 (2)	94 (0.5)	52 (0.5)	72 (0.5)	40	72 (5)
	R: 5′-GCTGTTAGTGCCTAAAGAAGCAT-3′							
m2	F: 5′-GGAGCCCCAGGAAACATTG-3′	352	95 (2)	94 (0.5)	52 (0.5)	72 (0.5)	40	72 (5)
	R: 5′-CATAACTAGCGCCTTGCAC-3′							
cagA	F:5′-TTGACCAACAACCACAAACCGAAG-3′	183	94 (2)	95 (0.5)	50 (0.75)	72 (0.75)	40	72 (5)
	R:5′-CTTCCCTTAATTGCGAGATTCC-3′							

Detection of *vac*A alleles and *cag*A. The *vac*A and *cag*A genotyping was performed by PCR by using specific primers, as described in Table 1. PCR was conducted in 25-μL volumes containing 1 × PCR buffer, 1 μL of the DNA template, 0.2 mmol/L dNTP mixes, 6 pmol of each primer, and 0.75 U Taq polymerase (New England Biolabs, Ipswitch, MA, USA). The amplified products were electrophoresed on a 1.5% agarose gel, stained with ethidium bromide, and visualized under ultraviolet illumination.

### Immunization of dairy cows

The Animal Care Committee of Hebei Academy of Agriculture and Forestry Sciences approved the immunization protocol. Four pregnant dairy bovines were vaccinated with the mixture of Hp5162 and Hp4236 *H. pylori* for a total of 8 times, 2 times during the last month before parturition, and 6 times after parturition. Vaccination was performed each time with 6 × 10^9^ cfu *H. pylori*, by means of intranasal mucosal drip, supramammary lymph node (subcutaneous), and hip muscle administration at four sites (once every 2 weeks). Dairy cows were boosted at about 2-week intervals. The *H. pylori* suspension was emulsified with an equal volume of Freund’s complete adjuvant for the initial immunization and Freund’s incomplete adjuvant for each of the boosts. Milk and serum samples were collected from each cow before immunization and 50 days after immunization for the detection of antibodies by ELISA.

### Detection of antibody by ELISA

Overnight cultures of bacteria were collected by centrifugation (5,000×*g*, 10 min, 4°C), washed three times with PBS, and repelleted at 10,000×*g* for 2 min. Total proteins were extracted by using the ProteoPrep^®^ Sample Extraction kit (Sigma-Aldrich Corporation, St. Louis, MO, USA) according to the manufacturer’s instructions. The protein concentration was measured by the Pierce™ BCA Protein Assay kit (Thermo Fisher Scientific Inc, Waltham, MA, USA). Microplates were pretreated with 2.5% glutaraldehyde, and each ELISA plate was coated with 100 µl *H. pylori* at a concentration of 1 mg/mL in PBS and incubated overnight at 4°C. The coated wells were blocked with PBS containing 2.5% bovine serum albumin (Sigma-Aldrich Corporation, St. Louis, MO, USA) for 2 h at 37°C. A 100 µL milk sample, which was diluted 1:200, 1:400, 1:800, and 1:1,200 with PBS containing 0.05% Tween 20 was dispensed to the microplate. Horseradish-peroxidase-conjugated goat anti-bovine IgG antibody (Southern Biotechnology Associates Inc, Birmingham, AL, USA) which was diluted 1:8,000 and tetramethylbenzidine peroxide substrate systems were used as the secondary antibody and the substrate, respectively. Absorbance at 450 nm (A450) was measured after the reaction was terminated with 1 M H_2_SO_4_. The maximal milk dilution giving an A450 of 0.5 was expressed as the antibody titer.

### Disinfection of milk

The presences of antibodies were confirmed by ELISA, and the antibody titer attained a peak (1:800) by serial detection. The cow antibody-containing milk was then collected, defatted, processed into sterile milk by pasteurization, and stored at −20°C until required. Similarly, milk prepared from non-immunized cows was used as the placebo.

### Clinical trial

The study protocols were approved by the Ethics Committee of Hebei Medical University Fourth Hospital according to Declaration of Helsinki criteria. All subjects were provided with a notice of written consent before enrollment. Because of the exploratory nature of this study, the sample calculation was based on previous literature of about 1–5%/year spontaneous clearance rate of *H. pylori* infection [34]. Based on Fisher’s exact test with a unilateral hypothesis and an expectation of an increase in the eradication rate of 30% with bovine anti-*H. pylori* antibodies compared with the 5%/year spontaneous clearance rate of the infection, the sample size was calculated to be 28 patients for each group of either the antibody milk or placebo group for a power of 0.80 and a significance level of 0.05. The clinical study was designed to be a randomized double-blind placebo-controlled trial. The randomization was carried out by using a list obtained by computerization. Patients received their numbers in an ascending order for enrollment. This number corresponded to the randomized regimen for the use of medication with antibody milk or placebo. None of the patients were aware of the randomization, and the investigators, who were also blinded to the randomization, followed the treatment and performed all examinations independently.

416 volunteers from Shijiazhuang city were enrolled for the trial, the volunteers population consisting of 233 men and 183 women with a mean age of 39 (age range 20–67). First, the patients infected with *H. pylori* were screened by detecting anti-*H. pylori* IgG antibody in the volunteer’s serum by using the ASSURE *H. pylori* Rapid Test kit (MP Biomedicals LLC, Santa Ana, CA, USA). Second, volunteers with positive serum antibodies were confirmed as being infected with *H. pylori* by the carbon-14-labeled urea breath test. According to the inclusion and exclusion criteria, 61 volunteers (34 males and 27 females) were enrolled in the clinical trial and randomly assigned to either the antibody milk (in total 31 subjects, 15 males and 16 females, age range 21–57, mean 36) or placebo (in total 30 subjects, 19 males and 11 females, age range 26–63, mean 42) group. *Inclusion criteria were* subjects who were enrolled were over 18 years old with *H. pylori* infection and the O blood group, with no previous treatment for the infection and no use of anti-inflammatory or antibiotic drugs within 4 weeks prior to enrollment. *Exclusion criteria were* subjects were excluded with lactose intolerance, pregnancy or lactation, malignancy, significant systemic comorbidity, over 80 years, a history of gastrointestinal surgery, erosive esophagitis, or use of low-dose aspirin. Patients who had undergone previous treatment for *H. pylori* infection with standard therapy were also excluded.

The stored cow milk was thawed and warmed at 37°C prior to use. Based on the recommended daily amount of the consumption of milk, all subjects received either 200 mL immune-milk + 1 mL NaHCO_3_ (1 mol/L) or placebo + 1 mL NaHCO_3_ (1 mol/L) once a day, 1–1.5 h after dinner for 28 days. The follow-up period was 56 days. ^14^C-urea breath tests were performed three times, namely at the start of the study, after completion of the study medication, and at the end of the follow-up. The questionnaires for adverse events were completed at the 7, 14, 28, and 56 days; all patients answered a questionnaire on dyspeptic symptoms and the most common symptoms related to possible adverse effects attributable to treatment (Table 2). Each symptom was quantified as –(0), +(1), ++(2), and +++(3). The questionnaire allowed the inclusion of new symptoms that were considered as adverse effects in the assessments after antibody milk administration. The number and grade of symptoms in all patients were evaluated. Previous symptoms that increased in grade during and after treatment were also considered as adverse effects.

**Table 2:** Symptom questionnaires

Symptom	–	+	++	+++
Flatulence				
Abnormal taste				
Diarrhea				
Vomiting				
Epigastric pain				
Pirosis				
Heartburn				
Regurgitation				
Postprandial fullness				
Nausea				
Other (discriminate)				
Values	0	1	2	3

+++ Symptom interferes with normal activity >50% of evaluated time, ++ symptom interferes with normal activity <50% of evaluated time, + symptom does not interfere with normal activity, – without symptoms.

### Data analysis

A statistical analysis was performed by using SPSS 17.0 software for Windows. The Pearson Chi Square test was used to compare variables for clearance and adverse effect incidence, and a two-sided P < 0.05 was considered statistically significant. The grades of adverse events at 7, 14, 28, and 56 days were evaluated by the Mann–Whitney test by using the value obtained from the symptom questionnaire.

## Results

**Figure 1:**
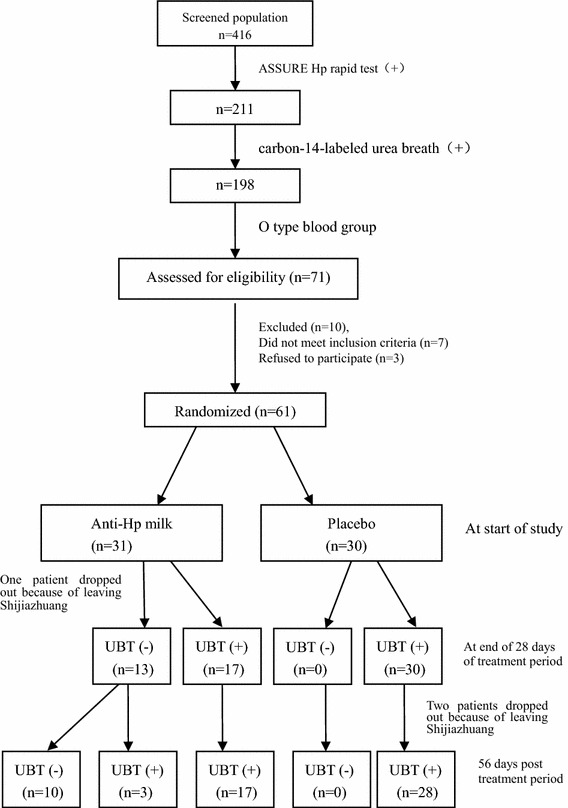
Patient flow in the pilot study with bovine anti-*Helicobacter pylori* antibodies in milk.

**Figure 2:**
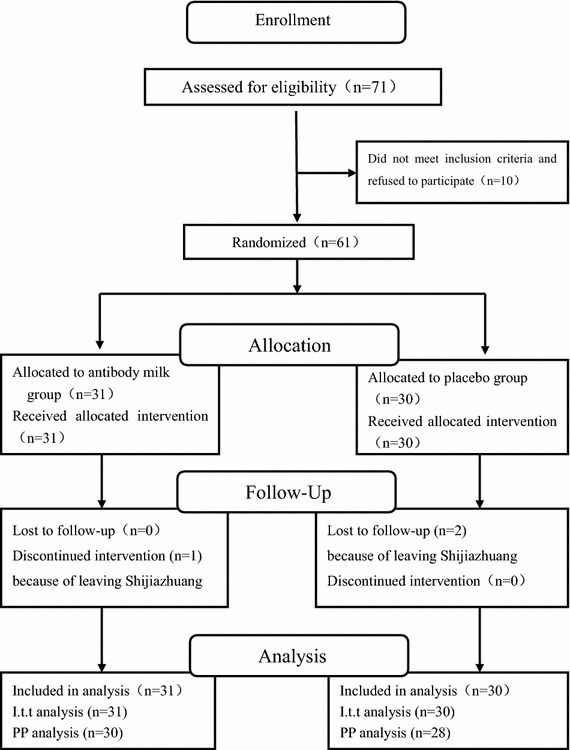
Consort diagram. *ITT* intention to treat, *PP* per protocol.

One volunteer in the antibody group and 2 volunteers in the control group dropped out of the trial because they had moved out of the region; 58 subjects completed the study with 30 in the antibody group and 28 in the placebo group (Figures 1, 2). The clearance efficacy of the antibody milk was evaluated according to the results of the ^14^C-urea breath tests; 13 of 30 antibody-treated subjects became UBT negative, whereas none of the 30 placebo-treated subjects became UBT negative after completion of the study medication; 3 of the 13 UBT-negative subjects became positive at the end of the follow-up. The per-protocol (PP) and intention to treat (ITT) with antibody milk and with placebo both indicated a significant difference in the clearance rate of infected patients between the bovine milk containing antibodies and the placebo-treated group (P = 0.001, P < 0.05). No significant differences were seen in the score of adverse effect in the antibody group compared with the placebo group on 7, 14, 28, and 56 days (P > 0.05 for all).

### Safety

None of the patients experienced adverse effects attributable to the antibody milk treatment, and the antibody milk was well tolerated.

## Discussion

The previous in vitro studies have demonstrated the good effects of treatment with bovine antibody-containing milk, but the in vivo clinical studies that are available have shown no *H. pylori* eradication [22–29, 35, 36]. More studies on a variety of factors such as the optimal length of treatment, the optimal antibody dose, adjunctive acid suppression, are needed to solve the discrepancy of outcome between the in vitro and in vivo investigations.

*Helicobacter pylori* inhabits the 100 µm-thick mucus layer, particularly the 25 µm close to the gastric epithelial cells [37]. Previous literature has shown that pH influences the extent of binding of *H. pylori*-specific antibodies in the milk to *H. pylori*, with no binding being observed under pH 4.0 and maximum binding at pH 7–8 [38]. Thus, NaHCO_3_ has to be added to the milk so that the elevation in mucus pH can decrease the viscosity of the mucus and contribute to the penetration of antibodies into the mucus.

The main problems with the use of antibodies to prevent and control infectious diseases are the variability of the antigen. The genotype and serotype of *H. pylori* of different regions varies significantly from country to country, and even between regions within the same country [39, 40]. Previous studies have indicated that the *H. pylori*-specific antibodies bind to different clinically isolated *H. pylori* stains with less than a tenfold variation in titer [38]. Our clinical trial has shown a positive effect and safety in O blood group *H. pylori*-infected subpopulations following the oral administration of polyclonal antibodies in defatted milk, in which casein has not been discarded considering that casein can elevate mucus pH. However, to date, an effective treatment of the *H. pylori* infection has proven difficult, considering costs, adverse effects, and the increasing emergence of antibiotic-resistant strains [12, 13, 15, 41, 42]. The use of anti-*H. pylori* bovine antibodies in milk to control the *H. pylori* infection has many advantages such as low costs, nutrients, and good compliance, without the development of antibiotic resistance in *H. pylori* or other flora and the tolerance of long-term use. Many factors might influence the clinical effect, including the different pH microenvironments between patients, the antibodies titers in the milk, high gastric acidity, a high bacterial load, persistent infection, bacterial self-protecting mechanisms (such as the flagella of *H. pylori*, which are covered by a flagellar sheath and therefore are thought to be shielded from antibody selection), the different blood types of patients, the genotype deference between immunized and infected bacteria, the optimal length and dose of treatment, the oral administration time, and the size of the study population. However, the degree of match between the immunization and the patient infection strains might be the crucial in influencing the effect of the clinical trial. Therefore, we have selected the locally prevalent *H. pylori* stains to immunize the dairy cows. *H. pylori* possesses an enormous genomic diversity and plasticity that facilitate host adaptation. Simultaneously, many studies have suggested that *H. pylori* has a much higher recombination rate and mutation than most other microorganisms [43]. Variable expression of fucosyltransferases enables *H. pylori* strains to alter their host-recognizable antigens by mimicking human Lewis antigens in order to evade their host’s immune response [9, 44]. Genetic diversification of the *H. pylori* adhesin genes might allow the adaptation of adherence properties to better facilitate persistence, despite host defenses. The different genotypes of *H. pylori* have their respective phenotypes with regard to cell envelope and surface structure, especially their adhesins [45]. Different adhesins bind different antigens [46, 47]. For example, adhesins SabB and SabA bind sialic acid, and adhesin BabB binds Lewis b antigen [47, 48]. Frequent *H. pylori* infection in crowded living conditions and the clustering of the cases occurring within family units indicate that different regions have different locally prevalent strains. The local prevalent *H. pylori* strains must therefore be selected to immunize the dairy cows. However, the overall efficacy of the 33.3% (10/30) clearance rate in our study is low and is not suitable clinically for the treatment of *H. pylori* infection. This might be because the selected immunization strain was not the actual locally prevalent strain in our study because we screened a single *vac*A gene locus. More gene loci such as the genes of the flagellum and adhesins need to be screened.

Breast-feeding during the first months after birth decreases human infant morbidity and mortality from diarrheal and systemic infection because the colostrum conveys protection to the immunologically naïve infant against a variety of microbial pathogens by immunoglobulins [49]. Whole bovine immune-milk mimics natural protection in humans and has been used in infants for the treatment or prevention of enteric infections by bacterial, viral, and protozoal pathogens. *H. pylori* expresses adhesins of BabA/B, SabA, AlpA/B, OipA, and HopZ, which confer intimate adherence to the gastric epithelium, whereby the bacteria can gain easy access to nutrients from host tissues [10, 11, 37, 46, 48]. Antibodies against BabA/B, SabA/B, AlpA/B, OipA, HopZ, and urease can disrupt the ability of *H. pylori* to colonize the gastric mucosa and limit its ability to garner essential nutrients. Our study might have been limited by the fact that we did not ascertain whether milk taken from immunized cows contained the specific antibodies mentioned above. Therefore, future research is necessary to ascertain the type of antibodies described above.

At the end of the follow-up, 3 of the 13 UBT-negative patients relapsed partly because of the existence of bacteria in the intercellular junctions or the intracellular submucosa which the bovine antibodies were unable to reach in sufficient concentrations to block the bacteria. Recent studies have indicated that the oral cavity is not only a potential reservoir for *H. pylori* infection of the stomach, but also a potential reservoir for re-infection. Previous studies have indicated that patients with oral *H. pylori* are at a significantly greater risk of gastric reinfection following successful therapy [50, 51]. Another explanation is that mixed infections by two or more strains are common in China [52].

## Conclusions

Although the bovine antibody-based oral immunotherapy demonstrated good effects on controlling the *H. pylori* infection in our study, it cannot fully replace the therapeutic strategies with antibiotics, because of the small size of the investigated population. Further studies on larger populations and the relationships between the genetic diversity and pathogenicity of *H. pylori* strains, an analysis of the volunteers’ *H. pylori* genotype and phenotype, and the selection of the actual locally prevalent *H. pylori* stains will be required before this particular novel and potentially therapeutic strategy for the eradication of this microorganism can be fully developed.

## Abbreviations

cagA: cytotoxin associated gene A
vacA: vacuolating cytotoxin A
BabA/B: blood group antigen-binding adhesion A/B
SabA/B: sialic acid-binding adhesion A/B
HopZ: Helicobacter outer membrane porins Z
AlpA/B: adherence-associated lipoprotein A/B
OipA: outer inflammatory protein A
